# Multimodal Physiological Monitoring Using Novel Wearable Sensors: A Pilot Study on Nocturnal Glucose Dynamics and Meal-Related Cardiovascular Responses

**DOI:** 10.3390/bioengineering13010069

**Published:** 2026-01-08

**Authors:** Emi Yuda, Yutaka Yoshida, Hiroyuki Edamatsu, Junichiro Hayano

**Affiliations:** 1 Innovation Center for Semiconductor and Digital Future (ICSDF), Mie University, Tsu 514-8507, Japan; 2 Department of Management Science and Technology, Graduate School of Engineering, Tohoku University, Sendai 982-0002, Japan; 3 School of Medicine, Nagoya City University, Nagoya 467-0001, Japan; yoshida@sda.nagoya-cu.ac.jp (Y.Y.); hayano@acm.org (J.H.)

**Keywords:** interstitial fluid glucose (ISFG), nocturnal hypoglycemia, wearable ring sensor, heart rate variability (HRV), remote health monitoring

## Abstract

This pilot study investigated multimodal physiological monitoring using minimally invasive and wearable sensors across two experimental settings. Experiment 1 involved five healthy adults (1 female) who simultaneously wore an interstitial fluid glucose (ISFG) sensor and a ring-type wearable device during sleep (00:00–06:00). Time-series analyses revealed that ISFG levels decreased during sleep in four of the five participants. ISFG values were significantly lower in the latter half of the sleep period compared with the first half (0–3 h vs. 3–6 h, *p* = 0.01, d = 2.056). Four participants also exhibited a mild reduction in SpO_2_ between 03:00–04:00. These results suggest that nocturnal ISFG decline may be associated with subtle oxygen-saturation dynamics. Experiment 2 examined whether wearable sensors can detect physiological changes across meal-related phases. Nine male participants were monitored for heart rate (HR) and skin temperature during three periods: pre-meal (Phase 1: 09:00–09:30), during meal consumption (Phase 2: 12:30–13:00), and post-meal (Phase 3: 13:00–13:30). A paired comparison demonstrated a significant difference in median HR between Phase 1 and Phase 2 (*p* = 0.029, d = 0.812), indicating a large effect size. In contrast, HR–temperature correlation was weak and not statistically significant (Pearson r = 0.067, *p* = 0.298). Together, these findings demonstrate that multimodal wearable sensing can capture both nocturnal glucose fluctuations and meal-induced cardiovascular changes. This integrative approach may support real-time physiological risk assessment and future development of remote healthcare applications.

## 1. Introduction

Continuous monitoring of glucose dynamics is of great clinical and physiological importance, particularly in detecting subtle metabolic changes that may not be captured by routine testing. Interstitial fluid glucose (ISFG) monitoring has emerged as a minimally invasive approach that enables dynamic assessment of glucose fluctuations in real-world and clinical settings. Over the past two decades, numerous studies have explored ISFG monitoring in a wide range of contexts [1,2,3,4,5,6,7,8,9]. Research has extensively examined postprandial glucose responses to dietary intake [1,2], as well as applications in patients undergoing dialysis, where glycemic control is often complicated by altered metabolic homeostasis [3,4]. In addition, ISFG monitoring has been integrated into broader metabolic and lifestyle research, including exercise physiology, stress-related responses, and circadian rhythm assessments [5,6,7,8,9]. Collectively, these findings underscore the versatility of ISFG monitoring as a tool for capturing physiologically relevant glucose fluctuations.

One particularly critical application of ISFG monitoring is the detection and characterization of nocturnal hypoglycemia. Nocturnal hypoglycemia is often asymptomatic and thus difficult to recognize in routine care, yet it is associated with serious acute and long-term consequences, including cardiovascular stress, impaired neurocognitive function, and increased mortality risk. In recent years, this condition has received growing attention in both basic and clinical research. Studies have proposed predictive and detection technologies that leverage continuous glucose monitoring (CGM), machine learning algorithms, and multimodal sensor integration to improve recognition of nocturnal hypoglycemia in real time [10,11,12,13,14,15,16,17,18,19,20,21]. Parallel to these technological advances, clinical investigations have emphasized the impact of nocturnal hypoglycemia on patient management, with specific focus on treatment intensification, individualized insulin titration, and patient education strategies [22,23,24,25,26,27,28,29,30,31,32]. Furthermore, therapeutic innovations such as modified insulin formulations, adjunct pharmacotherapies, and tailored behavioral interventions have been explored to reduce the risk of nocturnal hypoglycemic episodes [33,34,35,36,37,38,39,40].

Beyond applied technologies and clinical interventions, basic research has provided essential insights into the physiological mechanisms underlying nocturnal hypoglycemia. Several studies have highlighted the role of cortisol secretion patterns, which may contribute to early-morning glucose nadirs and altered counterregulatory responses [41,42,43,44,45,46,47]. Others have focused on the autonomic nervous system, where reduced sympathetic activation during sleep may impair glucose counter regulation and exacerbate the risk of hypoglycemia [42,47]. At a broader level, foundational work in pathophysiology and general conceptual frameworks has contextualized nocturnal hypoglycemia within the dynamics of circadian biology, endocrine regulation, and systemic energy balance [41,42]. Together, these studies emphasize that nocturnal hypoglycemia is a multifactorial condition, requiring a multimodal and interdisciplinary approach to monitoring, prediction, and intervention. In parallel, rapid advances in flexible electronic devices, particularly MXene-based sensors, are expanding possibilities for next-generation wearable health monitoring [48].

Despite the considerable progress in continuous glucose monitoring technologies and the expanding landscape of multimodal sensing, several important gaps remain in understanding how glucose dynamics interact with other physiological parameters under naturalistic conditions. In particular, the relationship between nocturnal glucose fluctuations and concurrent cardiorespiratory changes has not been fully elucidated. While prior studies have explored the detection of nocturnal hypoglycemia, few investigations have simultaneously captured interstitial glucose levels together with peripheral physiological signals such as heart rate, oxygen saturation, and movement during sleep. Integrating ISFG measurements with wearable-derived physiological indices may offer new insights into how subtle alterations in autonomic regulation or oxygenation accompany nocturnal glucose reductions—especially in otherwise healthy individuals who may exhibit unrecognized metabolic variability.

Furthermore, daily glucose and metabolic regulation are strongly influenced by meal-related physiological responses. Although postprandial glucose dynamics have been widely studied, much less is known about whether wearable sensors can reliably detect concurrent cardiovascular or thermoregulatory changes across pre-meal, meal, and post-meal phases. Heart rate and skin temperature are especially relevant, as they reflect metabolic workload and peripheral blood flow redistribution, respectively. Understanding whether these parameters shift in predictable ways in response to food intake—and whether such shifts can be sensitively detected using noninvasive wearable devices—may support the development of real-time metabolic assessment tools.

To address these gaps, the present pilot study comprised two complementary experiments with distinct methodological aims. Experiment 1 was designed as an exploratory feasibility study rather than a formal hypothesis-testing investigation. Specifically, it sought to evaluate whether nocturnal trends in interstitial fluid glucose (ISFG) could be reliably captured during free-living sleep conditions using minimally invasive glucose sensing in combination with wearable-based physiological monitoring. Expectations, rather than predefined hypotheses, guided this experiment, with exploratory and secondary statistical analyses used to characterize potential associations. The primary focus was on assessing the feasibility of concurrently recording nighttime ISFG dynamics alongside heart rate (HR), peripheral oxygen saturation (SpO_2_), and actigraphy signals, and on examining whether temporally aligned fluctuations across these modalities could be observed. From a physiological perspective, nocturnal declines in glucose levels may coincide with transient alterations in autonomic nervous system activity and oxygenation status during sleep. Prior studies have suggested that shifts toward parasympathetic dominance, sleep-stage transitions, or brief arousal-related events can influence both cardiovascular regulation and peripheral oxygen saturation, potentially interacting with glucose homeostasis through neuroendocrine and metabolic pathways. Experiment 1 therefore explored whether such physiological signatures could be detected in conjunction with nighttime ISFG changes, without presuming causal relationships. Experiment 2, in contrast, was structured to evaluate the capacity of wearable sensors to detect meal-induced cardiovascular and thermoregulatory responses under controlled daily-life conditions. Heart rate and skin temperature were measured across pre-meal, meal, and post-meal phases to assess wearable sensitivity to acute metabolic challenges.

## 2. Materials and Methods

### 2.1. Experiment 1 Participants

Five healthy volunteers (1 female, mean age 55 ± 10 years) were recruited for this study. None of the participants reported a history of metabolic, cardiovascular, or sleep disorders, and none were taking medications that could affect glucose metabolism or autonomic function. Written informed consent was obtained from all participants prior to enrollment. The study protocol was reviewed and approved by the Ethics Committee of the Graduate School of Engineering, Mie University (Approval number: 132, Approval date: 19 February 2025) and the Ethics Review Committee of Nagoya City University Hospital (Approval number: 60-18-0211, Approval date: 22 March 2019). All procedures were conducted in accordance with the Declaration of Helsinki.

### 2.2. Sensors and Data Acquisition (Experiment 1)

Interstitial Fluid Glucose (ISFG) Monitoring; a minimally invasive interstitial fluid glucose (ISFG) sensor (FreeStyle Libre, Abbott, Abbott Park, IL, USA) was attached to the upper arm and continuously recorded glucose concentrations at 15-min intervals over a 14-day monitoring period. Periods of missing or unreliable glucose data—arising from sensor detachment, temporary signal loss, or device warm-up and calibration phases—were identified using manufacturer-provided quality flags and time stamps.

For data handling, short gaps in ISFG data (<30 min, corresponding to ≤2 consecutive missing samples) were linearly interpolated for visualization purposes only. Longer gaps were excluded from all analyses. No imputation was applied for exploratory or inferential statistical analyses to avoid introducing artificial temporal structure into the glucose signal.

**Figure 1:**
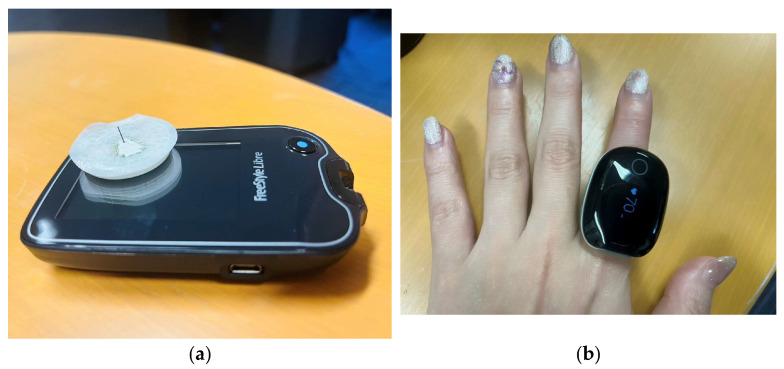
Continuous monitoring of interstitial fluid glucose levels. (**a**) Continuous Glucose Monitoring (CGM) or Intermittently Scanned CGM (isCGM):A sensor-based device designed for diabetes management that eliminates the need for conventional finger-prick blood glucose testing. By simply scanning a patch-type sensor worn on the arm with a dedicated reader or smartphone, users can check interstitial fluid glucose levels at any time. The device operates via a fine, flexible filament inserted under the skin, which records glucose concentrations by converting enzymatic reactions into electrical signals. It should be noted that there is typically a 5 to 15-min time lag compared to actual blood glucose levels, as it takes time for glucose to move from the blood into the interstitial fluid. (**b**) Ring-type Pulse Oximeter:A wearable device worn on the finger to continuously monitor pulse rate and oxygen saturation (SpO_2_) during sleep. It is specifically optimized for screening Sleep Apnea Syndrome (SAS) and monitoring overall sleep quality. Compared to wrist-worn devices, the ring design provides higher stability at the base of the finger, reducing motion artifacts and physical discomfort during rest. A key feature is the vibration alert function, which triggers when SpO_2_ drops below a preset threshold to encourage a change in breathing or body position. The device is capable of 10–12 h of continuous recording on a single charge. It utilizes a 3-axis accelerometer to detect body movements and saves data at 4-s intervals (0.25 Hz), an interval optimized to balance battery efficiency with the precise detection of significant respiratory events during sleep.

Physiological Signal Monitoring; physiological signals were acquired using a ring-type wearable device (Check-Me Ring, Sanei Medisys, Kyoto, Japan) worn on the index finger of the non-dominant hand (Figure 1). The device continuously recorded heart rate (HR), peripheral oxygen saturation (SpO_2_), and actigraphy.

Sampling and recording characteristics were as follows:

SpO_2_

Internal optical sensing: high-frequency PPG sampling (device-internal, proprietary);

Output and storage rate: 0.25 Hz (one value per second, internally averaged);

Heart rate (pulse rate);

Output and storage rate: 0.25 Hz.

Actigraphy (motion)

Output and storage rate: 0.25 Hz (one value every 4 s), configured as a low-power setting optimized for detecting gross movements such as postural shifts and body turns during sleep.

Physiological data were transmitted to a smartphone via Bluetooth Low Energy (BLE 4.0) using the manufacturer’s application and exported as comma-separated value (CSV) files for offline analysis. Manufacturer-specified measurement ranges were 70–99% for SpO_2_ and 30–250 bpm for HR, with stated accuracies of ±2% for SpO_2_ (80–99%), ±3% for SpO_2_ (70–79%), and ±2 bpm or ±2% (whichever was greater) for HR.

(i) Criteria for Retaining or Excluding Sleep Data Segments

Primary analyses focused on the habitual nighttime interval between 00:00 and 06:00, based on participant self-reports and actigraphy-derived rest–activity patterns. Sleep data segments were retained for analysis only if all of the following conditions were met:

Concurrent availability of ISFG, HR, and SpO_2_ data;

Absence of prolonged missing data (>30 min) in any modality;

Low actigraphy levels consistent with sleep or minimal movement.

Segments were excluded if sustained high actigraphy suggested wakefulness, device manipulation, or substantial finger motion. This approach prioritized temporal overlap and data integrity rather than attempting formal sleep staging.

(ii) Motion Artifact Detection and Signal Quality Filtering

To mitigate motion-related artifacts and non-physiological signal contamination, a rule-based filtering framework was applied prior to analysis:

SpO_2_-based exclusion

Values outside the measurable range (70–99%) were discarded;

Abrupt, isolated drops inconsistent with adjacent values were flagged when temporally coincident with elevated actigraphy.

Heart rate-based exclusion

Data points were excluded if HR changed by more than a physiologically implausible threshold within a single 1-s interval (e.g., >30 bpm/s).

Actigraphy-based exclusion

Periods of sustained elevated motion (0.25 Hz signal indicating continuous movement over multiple samples) triggered exclusion of overlapping HR and SpO_2_ segments.

After automated filtering, remaining signals were visually inspected. A short moving-average filter was applied to HR and SpO_2_ time series to reduce high-frequency noise while preserving low-frequency nocturnal trends. Filtering parameters were chosen conservatively to avoid distorting temporal patterns of interest.

(iii) Temporal Alignment and Synchronization Across Sensors

Temporal synchronization across ISFG and wearable-derived physiological signals was performed using device time stamps. Given differences in sampling frequency, multimodal alignment was achieved by aggregating HR and SpO_2_ data to match the 15-min ISFG intervals using median values within each window.

A temporal alignment tolerance of ±60 s was considered acceptable for synchronizing physiological data streams. Segments exceeding this tolerance due to clock drift, transmission delays, or missing data were excluded from multimodal analyses. This tolerance was selected to balance temporal precision with practical constraints inherent to consumer-grade wearable devices.

Experimental Context and Analytical Scope

Participants were instructed to maintain their habitual sleep routines and to refrain from alcohol consumption or strenuous exercise on the day of measurement. No strict standardization of dietary intake, meal timing, or sleep environment was imposed in order to preserve ecological validity.

As a result, inter- and intra-individual variability in nocturnal glucose and physiological signals likely reflects a combination of behavioral factors, endogenous variability, and sensor-related characteristics. These factors were explicitly considered during interpretation. Accordingly, the analyses were designed to characterize data quality, temporal patterns, and multimodal feasibility rather than to infer controlled physiological mechanisms or causal relationships.

Figure 1a shows a sensor used to measure glucose levels in interstitial fluid. It comes with an ultra-fine filament (needle) that is inserted into the skin during application. This is not designed to puncture the skin or leave the needle in place; instead, it is inserted into the skin’s interstitial spaces to measure glucose concentration in the interstitial fluid. The filament reacts with glucose in the interstitial fluid using an enzyme inside the sensor, and the resulting weak electrical current is measured. The strength of this current is used to calculate blood glucose levels. Since there is no need to prick the fingertip with a needle, this method offers the advantage of reduced pain and discomfort compared to traditional blood glucose meters. Figure 1b shows the measurement using a ring-shaped sensor.

### 2.3. Data Processing and Analysis (Experiment 1)

All physiological data were synchronized by timestamp. ISFG measurements were linearly interpolated to generate a continuous time series at 0.25 Hz, matching the sampling rate of the ring-type device. HR, SpO_2_, and actigraphy signals were used as recorded, without filtering or smoothing.

The actigraphy signal was analyzed to confirm sleep onset and identify periods of excessive movement. Datasets with substantial motion artifacts during the target sleep interval were excluded. Resultant acceleration was calculated from the three-axis accelerometer embedded in the ring device, using:

(1) $$a_{mag}=\sqrt{a_{x}^{2}+b_{y}^{2}+c_{x}^{2}}$$

(2) $$\theta =arccos\left(\frac{a_{z}}{\sqrt{a_{x}^{2}+a_{y}^{2}+a_{z}^{2}}}\right)$$

where *a_x_*, *a_y_*, and *a_z_* represent accelerations along the three axes (units: g). This measure reflects changes in overall acceleration magnitude. Body position was further inferred from the tilt angle (*θ*), calculated as the deviation of the vertical (Z-axis) component from gravity:

*θ* ≈ 0°: Standing.

*θ* ≈ 90°: Supine, prone, or lateral recumbent (lying down).

The horizontal components (*a_x_*, *a_y_*) were used to distinguish between supine, prone, and lateral postures. In this study, this classification was used primarily to confirm lying-down status during sleep.

For glucose analysis, mean ISFG levels were computed for two consecutive intervals: the first half of sleep (0–3 h) and the second half (3–6 h). Paired *t*-tests were applied to compare ISFG levels between intervals, with statistical significance set at *p* < 0.05. SpO_2_ values were averaged in 1-h bins, and visual inspection was used to identify common temporal patterns across participants. Statistical analyses were performed using SPSS version 28 (IBM Corp., Armonk, NY, USA).

### 2.4. Experiment 2 Participants

Nine healthy adult males (mean age ± SD: 66 ± 12 years) participated in Experiment 2. All participants had no history of cardiovascular disease and were not taking any medications on a regular basis. The study was conducted in accordance with the Declaration of Helsinki and approved by the Ethics Committee of the Graduate School of Engineering, Mie University (approval date: 17 October 2025; approval number: 135). All participants provided written informed consent prior to enrollment.

### 2.5. Sensors and Data Acquisition (Experiment 2)

Physiological data were collected using a wearable sensor device (silmee™ 22, TDK Corporation, Tokyo, Japan), which simultaneously records wrist skin temperature and heart rate. Measurements were obtained from the non-dominant wrist of each participant across three predefined phases: pre-meal (Phase 1: 09:00–09:30), during meal intake (Phase 2: 12:30–13:00), and post-meal (Phase 3: 13:00–13:30).

Meal timing and synchronization procedures; to ensure temporal alignment between physiological recordings and meal-related events, participants were instructed to begin food intake as close as possible to 12:30 and to complete the meal within the designated Phase 2 interval. The wearable device recorded physiological signals continuously across all phases using an internal clock synchronized to the participant’s smartphone at the start of the measurement day. Meal onset and completion times were logged by the experimenter and cross-checked with participant self-reports to confirm adherence to the scheduled time window. These time stamps were subsequently used to segment continuous physiological data into pre-meal, meal, and post-meal phases.

Participants were instructed to remain seated during Phase 2 and to minimize unnecessary upper-limb movement in order to reduce motion-related artifacts. Water intake during the measurement period was permitted but recorded. No additional constraints were placed on meal composition or caloric content, reflecting the study’s emphasis on ecological validity rather than tightly controlled metabolic testing.

This protocol was designed to capture autonomic and thermophysiological responses to a naturally occurring physiological load—food intake. Meal ingestion is known to induce metabolic activation, redistribution of blood flow, postprandial thermogenesis, and shifts in sympathetic–parasympathetic balance. Accordingly, this experiment enables assessment of (1) autonomic reactivity reflected in heart rate and (2) changes in heat production and peripheral blood flow reflected in wrist skin temperature.

The primary hypotheses were as follows:

**H1**  (Heart-rate increase during meal intake)**.** *Heart rate during Phase 2 is expected to be higher than during Phase 1, reflecting increased metabolic demand and autonomic activation associated with digestion.*

**H2**  (HR–temperature coupling)**.** *Because sympathetic activation during Phase 2 may redistribute blood flow toward the splanchnic circulation, peripheral wrist skin temperature changes are expected to be attenuated or transiently reduced compared with Phase 3, when postprandial thermogenesis and peripheral vasodilation may elevate skin temperature.*

### 2.6. Data Processing and Analysis (Experiment 2)

All data were processed and analyzed using Python 3.10.7. Heart rate (HR) and wrist skin temperature (Temp) recordings were synchronized and screened for missing or physiologically implausible values prior to analysis. Within-subject comparison (Paired *t*-test); to evaluate short-term autonomic responses to food intake, the median HR values in Phase 1 (P1) and Phase 2 (P2) were compared using a paired *t*-test. Repeated-measures ANOVA across phases; a one-way repeated-measures ANOVA was conducted with Phase (P1, P2, P3) as the within-subject factor to test for main effects of meal-related physiological load on HR. HR–Temperature association; to assess the coupling between autonomic activity and peripheral thermoregulation, all paired HR–Temp samples (across all participants and phases, excluding missing values) were pooled. The Pearson correlation coefficient (r) and corresponding *p*-value were computed to quantify the linear relationship between HR and wrist skin temperature. We adopted a significance level of *p* < 0.05.

## 3. Results

Exploratory nature of statistical analyses; all statistical analyses reported in the Results section, including *p*-values, were conducted within an explicitly exploratory and hypothesis-generating framework consistent with the pilot design of this study. Accordingly, *p*-values are provided to contextualize observed patterns and support descriptive interpretation, rather than to serve as confirmatory evidence. The results are therefore presented with an emphasis on descriptive trends, effect size estimates, and—wherever feasible—confidence intervals, and should not be interpreted as establishing definitive physiological relationships or causal effects.

### 3.1. Interstitial Fluid Glucose (ISFG) Dynamics During Sleep (Experiment 1)

**Figure 2:**
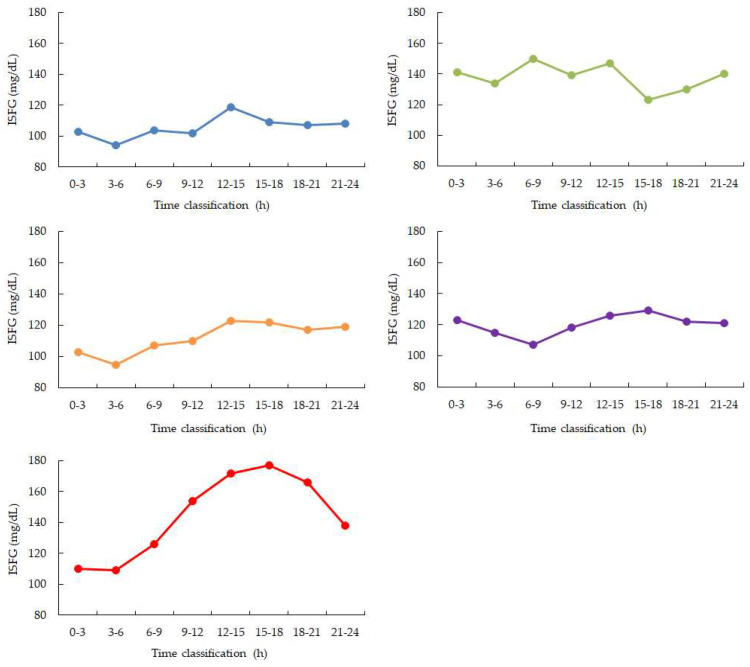
Diurnal variation calculated from 2 weeks of ISFG. Blue: participant 1, Green: participant 2, Orange: participant 3, Purple: participant 4, Red: participant 5. ISFG is measured as a 3-h average. During nighttime sleep, ISFG decreased in 4 out of 5 participants from 0:00 to 6:00. After that, ISFG increased in 4 participants (participants 1, 3, 4, and 5) from 12:00 to 15:00 and then gradually decreased throughout the night.

Between 00:00 and 06:00, four participants (Participants 1, 3, 4, and 5) showed a gradual and continuous decline in ISFG levels. In contrast, Participant 2 maintained relatively stable ISFG values without a clear downward trend (Figure 2, Table 1).

### 3.2. Peripheral Oxygen Saturation (SpO_2_), Heart Rate, and Motion (Experiment 1)

**Figure 3:**
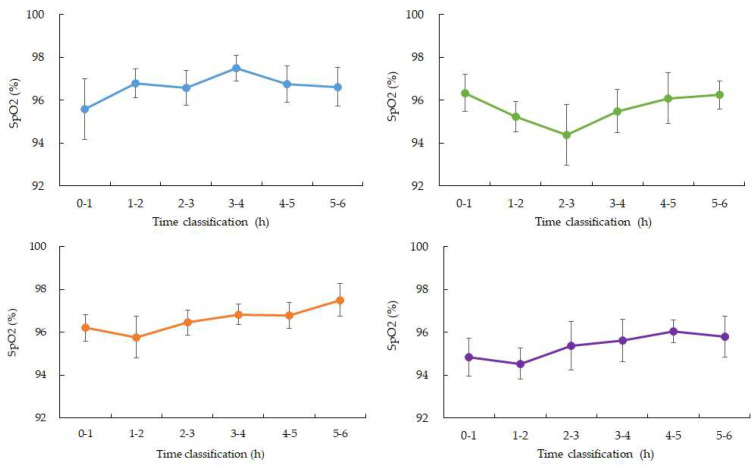
Changes in average SpO_2_ over time during nighttime sleep. Blue: participant 1, Green: participant 2, Orange: participant 3, Purple: participant 4. Hourly averages and standard deviations from 0 to 6:00 a.m. Participants 1, 3, and 4 showed a gradual increase from 3 to 4:00 a.m. after falling asleep. Participant 2, who had the highest ISFG among participants, showed a rapid decrease from 2 to 3:00 a.m. after falling asleep, and then recovered. (Data for participant 5 is missing).

SpO_2_ values were stable during the early sleep phases but showed a mild decline between 03:00 and 04:00 in four participants, with mean values decreasing by approximately 1–2% compared to earlier periods. Data from one participant were excluded due to device malfunction (Figure 2 and Figure 3). Heart rate showed the expected nocturnal slowing in all participants, with no arrhythmic events or abrupt fluctuations. Occasional minor HR increases coincided with motion artifacts, though large body movements were uncommon during the main sleep intervals (Table 2).

### 3.3. Quantitative Analysis (Experiment 1)

**Figure 4:**
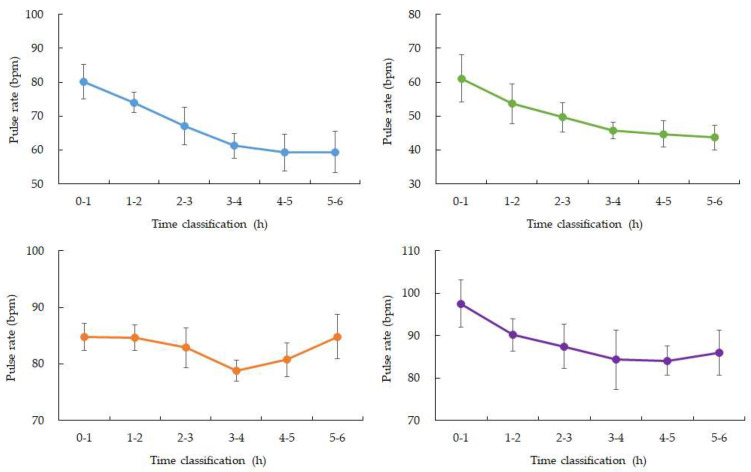
Changes in average SpO_2_ over time during nighttime sleep. Blue: participant 1, Green: participant 2, Orange: participant 3, Purple: participant 4. Hourly averages and standard deviations from 0:00 to 6:00. All four participants experienced a gradual decrease from 3:00 to 4:00 after going to bed.

Comparison of ISFG levels between the 0–3 h and 3–6 h intervals revealed a significant reduction in the latter (Figure 4). A paired *t*-test confirmed that this decrease was statistically significant (*p* = 0.01). The mean paired difference, defined as ISFG values during 0–3 h minus those during 3–6 h, was 7 mg/dL, with a 95% confidence interval of [3, 11] mg/dL. The effect size was large (Cohen’s dz = 2.056), indicating a robust decline in ISFG levels. Because interstitial glucose values are recorded with a resolution of 1 mg/dL, all glucose-related statistics are reported as integers (Table 3).

### 3.4. Cardiovascular and Thermophysiological Responses to Meal Intak (Experiment 2)

**Figure 5:**
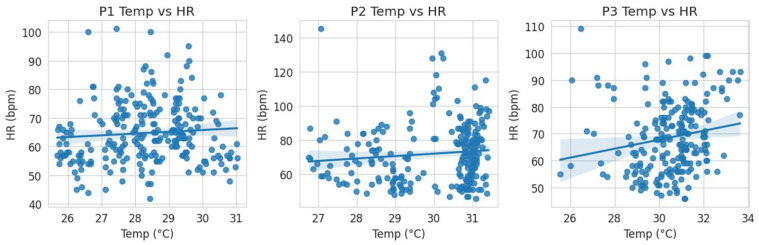
HR vs. Temp correlation (Pearson r and *p*-value).

The objective of Experiment 2 was to evaluate whether wrist-based measurements of heart rate (HR) and skin temperature could detect meal-induced autonomic and thermophysiological responses. Three analytical procedures were conducted: (1) a paired comparison of pre-meal and during-meal phases, (2) a repeated-measures analysis across all three phases, and (3) an assessment of the association between HR and skin temperature. Paired *t*-test (Phase 1 vs. Phase 2); a paired *t*-test was performed to compare the median HR between the pre-meal phase (P1) and the meal-intake phase (P2). The analysis revealed a significant increase in HR during meal intake (*p* = 0.029), indicating that digestion-related physiological load elicited a detectable autonomic response. The effect size was large (Cohen’s d = 0.812), supporting the robustness of the observed within-subject HR increase. Repeated-measures ANOVA across three phases; to evaluate HR differences across all three phases (P1, P2, P3), a repeated-measures ANOVA was conducted. The main effect of Phase was not statistically significant (F = 2.094, *p* = 0.151, η^2^ = 0.189). Although the effect size suggested a moderate proportion of variance explained, the absence of statistical significance indicates that the three-phase comparison did not yield clear systematic HR differences beyond the P1–P2 contrast identified in the paired *t*-test. Correlation between HR and wrist skin temperature; to examine the coupling between autonomic activation and peripheral thermoregulation, paired HR–temperature samples from all participants and phases were pooled. Pearson’s correlation analysis showed a very weak positive association (r = 0.067) that was not statistically significant (p = 0.298). These results indicate that, under the present conditions, wrist skin temperature did not covary meaningfully with HR during the meal-related physiological load (Table 4, Table 5 and Table 6, Figure 5).

The correlation between body surface temperature (Temp) and heart rate (HR) was analyzed for each phase (P1, P2, and P3). Phase-specific analysis showed a statistically significant positive correlation only in Phase P3 (r = 0.185, *p* = 0.006). No significant correlation was observed in Phase P1 (r = 0.079, *p* = 0.219) or Phase P2 (r = 0.107, *p* = 0.103). Comparisons of the correlation coefficients (r) between the phases were conducted using Fisher’s Z transformation test. No statistically significant difference was found when comparing the correlation coefficients between any of the three phases: P1 vs. P2 (Z = −0.309, *p* = 0.758), P2 vs. P3 (Z = −0.839, *p* = 0.402), and P1 vs. P3 (Z = −1.151, *p* = 0.250). The overall Pearson’s correlation coefficient between heart rate (HR) and body surface temperature (Temp) was 0.067 (*p* = 0.298). No significant correlation was detected in the pooled data.

## 4. Discussion

This pilot study examined multimodal physiological monitoring using two complementary paradigms: (1) nocturnal interstitial fluid glucose (ISFG) dynamics measured by continuous glucose monitoring (CGM) integrated with ring-type wearable sensing of heart rate, SpO_2_, and activity during free-living sleep, and (2) meal-associated physiological variations assessed using wrist-based heart rate and skin temperature measurements. The contribution of this work is incremental, and its novelty does not lie in the discovery of new physiological phenomena, but in the practical integration and temporal synchronization of CGM and wearable cardiovascular sensors under unconstrained, real-world conditions.

Previous CGM studies have largely examined glucose dynamics in isolation or under controlled settings, while wearable studies have typically focused on cardiovascular or activity signals independently. In contrast, the present study demonstrates the feasibility of synchronizing glucose, cardiovascular, and motion data streams during habitual nighttime sleep without imposing strict experimental control. This approach extends prior multimodal wearable research by incorporating continuous glucose monitoring into high–time-resolution wearable sensing, enabling joint observation of metabolic and physiological signals across nocturnal and daytime contexts.

Importantly, the observed temporal patterns across ISFG, heart rate, and SpO_2_ are descriptive and observational. No physiological coupling, regulatory mechanism, or causal relationship is inferred. Rather, this work provides a proof-of-concept demonstration that multimodal physiological signals can be acquired, aligned, and jointly interpreted in everyday environments, thereby establishing a methodological foundation for future hypothesis-driven and clinical investigations.

### 4.1. Interpretation of Experiment 1: Nocturnal Glucose and Physiological Dynamics

A consistent reduction in ISFG was observed in four out of five participants, with significantly lower values during the latter half of the sleep period (03:00–06:00) compared with the early sleep phase (00:00–03:00). This statistically significant difference (*p* = 0.01) indicates a reproducible temporal pattern in nocturnal ISFG levels across participants. Importantly, this observation is descriptive in nature and reflects an empirical characterization of glucose dynamics during sleep rather than evidence of altered metabolic demand or utilization.

Mild reductions in SpO_2_ were also observed around 03:00–04:00, remaining within physiologically normal ranges and temporally overlapping with the lowest ISFG values. This temporal co-occurrence does not imply a functional or physiological coupling between oxygen saturation and glucose dynamics. Rather, it illustrates that multiple physiological signals measured concurrently by wearable sensors can exhibit aligned timing patterns during sleep. Actigraphy and heart rate profiles confirmed stable sleep conditions during these periods, supporting the interpretation that the observed variations were not driven by motion or overt behavioral factors.

In addition to nocturnal patterns, a reproducible daytime elevation in ISFG was observed between 12:00 and 15:00. This finding is consistent with previously reported diurnal variability in interstitial glucose measurements and may reflect the combined influence of circadian timing, daily routines, or nutritional context. However, no attempt was made in the present study to disentangle or attribute these factors. Taken together, these results demonstrate the feasibility of continuous, multimodal monitoring to capture both nocturnal and diurnal glucose-related patterns, extending earlier ISFG studies that primarily focused on specific contexts such as meals [1,2], dialysis [3,4], device validation [5,6,7,8], or peri-operative settings [9]. The present findings should therefore be interpreted as hypothesis-generating observations rather than evidence of underlying physiological mechanisms.

### 4.2. Integration of Experiment 2: Meal-Related Autonomic and Thermophysiological Patterns

Experiment 2 explored whether wrist-based wearable sensors could capture observable physiological changes temporally associated with meal intake. A paired *t*-test indicated a significant increase in heart rate from the pre-meal phase (P1) to the meal intake phase (P2) (*p* = 0.029, d = 0.812). This result demonstrates a measurable difference in heart rate coinciding with the act of eating, without implying a specific autonomic mechanism or regulatory pathway.

In contrast, a repeated-measures ANOVA across all three phases (P1–P3) did not reveal a statistically significant overall phase effect (F = 2.094, *p* = 0.151), suggesting that the observed heart rate elevation was transient and most evident during active ingestion rather than sustained across the postprandial period. Furthermore, the correlation between heart rate and wrist skin temperature was weak and non-significant (r = 0.067, *p* = 0.298), indicating that concurrent temperature measurements did not systematically covary with heart rate under the present experimental conditions.

Collectively, these findings indicate that meal intake can be associated with detectable, time-locked changes in selected physiological signals as measured by consumer-grade wearable devices. When considered alongside the nocturnal observations in Experiment 1, the results support the feasibility of using the same wearable sensor platform to observe multimodal physiological patterns across distinct daily contexts, including sleep and meal-related activity. Crucially, the present study does not establish causal or functional relationships between glucose dynamics, cardiovascular responses, or thermoregulation. Instead, it demonstrates the practical capability of wearable sensors to simultaneously capture multiple physiological signals, providing a foundation for future hypothesis-driven investigations.

### 4.3. Methodological Contribution and Relation to Prior Work

The primary contribution of the present study lies in the technical integration of continuous interstitial glucose monitoring with ring-type wearable physiological sensing during free-living sleep, rather than in the demonstration of novel physiological mechanisms or clinical outcomes. While continuous glucose monitoring has been widely applied in both clinical and ambulatory contexts, prior CGM-focused studies have typically emphasized glycemic metrics in isolation or in combination with sparse contextual variables, such as self-reported sleep or activity. Conversely, wearable-based studies of heart rate, oxygen saturation, or actigraphy during sleep have often lacked concurrent metabolic measurements, limiting their ability to capture interactions between glucose dynamics and autonomic or cardiorespiratory regulation.

Existing efforts to combine CGM with wearable sensors have generally relied on wrist-worn devices, short laboratory protocols, or highly structured experimental conditions. As a result, a methodological gap remains regarding whether synchronized glucose, cardiovascular, and oxygenation signals can be stably acquired during unconstrained, habitual sleep using low-burden wearable form factors. Experiment 1 was specifically designed to address this gap at a pilot scale by evaluating the feasibility, signal alignment, and interpretability of nocturnal ISFG, HR, SpO_2_, and motion data collected simultaneously in a naturalistic home environment.

Within this framework, Experiment 2 serves a complementary role by demonstrating that the same class of wearable sensors can detect physiologically meaningful autonomic responses during a daytime metabolic challenge. Rather than functioning as an independent mechanistic study, the meal-related protocol provides a contextual validation of sensor responsiveness and temporal synchronization, reinforcing the interpretability of the nocturnal findings without imposing additional physiological claims.

Importantly, the present work does not seek to establish superiority over prior CGM or wearable approaches, nor to claim immediate clinical applicability. Instead, it addresses a specific and concrete methodological limitation in the existing literature: the scarcity of small-scale, proof-of-concept studies demonstrating synchronized, multimodal physiological data acquisition across both nocturnal and postprandial contexts under free-living conditions. By explicitly adopting an exploratory and feasibility-oriented design, this study illustrates how minimally invasive glucose sensing can be integrated with low-profile wearable devices to generate temporally aligned datasets suitable for subsequent hypothesis-driven research.

In this sense, the contribution of the present study is best understood as infrastructural rather than confirmatory. It provides empirical evidence that multimodal physiological integration is technically achievable outside laboratory settings and highlights practical considerations—such as data synchronization, artifact handling, and variability under naturalistic conditions—that are often underreported in larger-scale or more controlled studies. These methodological insights may inform future investigations aiming to develop robust, longitudinal monitoring frameworks for metabolic and autonomic function, particularly in populations where nocturnal glucose instability or autonomic dysregulation is of clinical concern.

### 4.4. Limitations and Future Directions

Several limitations should be acknowledged. Experiment 1 included only five participants, and Experiment 2 involved nine healthy older males, which limits generalizability across age groups, sexes, and metabolic conditions. In addition, the absence of polysomnography in Experiment 1 precluded precise temporal alignment of nocturnal ISFG patterns with sleep stages or arousal-related events. Moreover, nocturnal and condition-related variability was not systematically assessed, as measurements were limited to a single or limited number of nights per participant. As a result, the observed nocturnal ISFG patterns may be highly context-dependent and influenced by night-specific behavioral, environmental, or physiological factors. In Experiment 2, the lack of controlled energy intake and the absence of concurrent metabolic biomarkers restricted the interpretability of observed meal-associated cardiovascular and thermophysiological variations.

Importantly, both experiments were explicitly designed as pilot, feasibility-oriented investigations conducted under naturalistic conditions. The primary objective was to assess whether glucose monitoring could be temporally synchronized with wearable-derived physiological signals, rather than to test predefined mechanistic hypotheses or establish functional relationships. Accordingly, all statistical analyses were performed in an exploratory and hypothesis-generating context.

Emphasis was therefore placed on descriptive characterization of observed patterns, including central tendency measures, effect size estimates, and—wherever feasible—confidence intervals, to illustrate the magnitude and variability of signals captured by the multimodal system. Inferential statistics were used to contextualize these observations and support pattern identification, rather than to provide confirmatory evidence of physiological coupling or causality.

In particular, the findings from Experiment 1 should be interpreted as observational and exploratory. Although temporally coincident variations in nocturnal ISFG, heart rate, and SpO_2_ were observed, such temporal alignment does not imply causal, functional, or regulatory relationships among these signals. To minimize overinterpretation, *p*-values were considered alongside effect sizes and confidence intervals and were not treated as standalone indicators of physiological significance. Verification of these preliminary observations will require adequately powered, hypothesis-driven studies with rigorous physiological controls.

To evaluate the stability, reproducibility, and individual-level consistency of nocturnal glucose and physiological patterns, future studies will need to incorporate repeated measurements across multiple nights. Such longitudinal designs are essential for distinguishing night-to-night variability from stable individual characteristics and for assessing the robustness of observed multimodal trends under real-world conditions.

Future studies should involve larger and more diverse cohorts, incorporate polysomnography-validated sleep staging, implement standardized meal protocols, and include additional metabolic and autonomic biomarkers. Longitudinal measurements spanning multiple days and nights will be essential to assess intra-individual stability, reproducibility, and the robustness of observed multimodal patterns under real-world conditions.

Notably, the present study was not intended to directly compare the performance of single-sensor versus multimodal systems. Rather, its purpose was to demonstrate the feasibility, interpretability, and temporal alignment of metabolic and physiological signals obtained through the combined use of minimally invasive glucose sensing and wearable devices. To our knowledge, the integration of ISFG monitoring with wearable-derived heart rate, SpO_2_, motion, and skin temperature signals across both nocturnal and meal-related contexts represents an early proof-of-concept demonstration of synchronized multimodal physiological monitoring in unconstrained environments.

Taken together, these findings provide a methodological foundation for future mechanistic and clinical investigations. While confirmatory studies are clearly required—particularly for the exploratory observations obtained during sleep—the present work demonstrates the technical feasibility of integrated, real-time monitoring frameworks that may enable more comprehensive observational assessments of metabolic and physiological dynamics in everyday settings.

## 5. Conclusions

This study demonstrated the feasibility of multimodal, minimally invasive monitoring to observe metabolic and physiological signal patterns in real-world settings. During sleep, a reproducible nocturnal decline in ISFG was observed, while SpO_2_ remained largely stable and heart rate exhibited expected nocturnal slowing. These findings describe temporal characteristics of concurrently measured signals and do not imply underlying causal or mechanistic relationships. Experiment 2 further showed that the same sensing framework could capture time-locked physiological changes associated with meal intake, including a transient increase in heart rate from the pre-meal to the meal intake phase (*p* = 0.029, d = 0.812). However, comparisons across all phases using repeated-measures ANOVA were not statistically significant, and correlations between heart rate and skin temperature were weak and non-significant, indicating limited concordance among measured modalities under the present conditions.

Overall, the results indicate that an integrated wearable-based sensing approach can simultaneously capture nocturnal and postprandial physiological patterns. Rather than establishing functional or causal relationships, this work provides proof-of-concept evidence that multimodal physiological and metabolic signals can be temporally synchronized and observed using wearable technologies. Further validation in larger and more diverse populations is warranted to determine the robustness, reproducibility, and potential applications of this observational framework.

## Figures and Tables

**Table 1:** Average ISFG per time segment.

Participants	ISFG (mg/dL)	ISFG (0–3) *	ISFG (3–6)	ISFG (6–9)	ISFG (9–12)	ISFG (12–15)	ISFG (15–18)	ISFG (18–21)	ISFG (21–24)
1	106 ± 7	103	94	104	102	119	109	107	108
2	138 ± 8	141	134	150	139	147	123	130	140
3	112 ± 9	103	95	107	110	123	122	117	119
4	120 ± 6	123	115	107	118	126	129	122	121
5	144 ± 25	110	109	126	154	172	177	166	138

* The brackets in ISFG indicate time.

**Table 2:** Values obtained from biosensors during sleep (by time period).

Participants	HR (bpm)	HR (0–3a)	HR (3–6a)
1	67 ± 9	74 ± 7	60 ± 5
2	49 ± 7	54 ± 7	45 ± 4
3	83 ± 4	84 ± 3	81 ± 4
4	87 ± 6	90 ± 6	85 ± 6
	**SpO_2_ (%)**	**SpO_2_ (0–3)**	**SpO_2_ (3–6)**
1	97 ± 1	96 ± 1	97 ± 1
2	96 ± 1	95 ± 1	96 ± 1
3	97 ± 1	96 ± 1	97 ± 1
4	95 ± 1	95 ± 1	96 ± 1
	**Motion (G)**	**Motion (0–3)**	**Motion (3–6)**
1	0 [0-0]	0 [0-1]	0 [0-0]
2	0 [0-1]	0 [0-1]	0 [0-1]
3	0 [0-1]	1 [0-1]	0 [0-1]
4	0 [0-0]	0 [0-0]	0 [0-0]

Due to missing data, we were unable to obtain biosignal data during sleep from participant 5.

**Table 3:** Statistical Analysis of ISFG Scores and Paired *t*-Test Results.

Category/Metric	ISFG (0–3)	ISFG (3–6)	Difference/Statistics
Data 1	103	94	9
Data 2	141	134	7
Data 3	103	95	8
Data 4	123	115	8
Data 5	110	109	1
Mean	116.0	109.4	6.60 (Mean Diff.)
Standard Deviation (SD)	16.19	16.44	3.21
Standard Error (SE)	-	-	1.44
Pearson Correlation	-	-	0.981
Degrees of Freedom (df)	-	-	4
t-Statistic	-	-	4.60
*p*-value (Two-tailed)	-	-	0.01 *
95% Confidence Interval	-	-	[2.615, 10.585]
Effect Size (Cohen’s d)	-	-	2.056

The analysis shows a statistically significant decrease in ISFG scores (*p* = 0.01 < 0.05). Effect Size: A Cohen’s d of 2.056 represents a strong experimental effect. * *p* < 0.01 indicate significant differences.

**Table 4:** Heart Rate & Skin Temperature Across Phases.

Subject/Phase	Skin Temp Mean (°C)	Skin Temp SD	HR Mean (bpm)	HR SD	HR Missing Rate
Subject 1—P1	25.986	0.252	59.33	6.67	0.0%
Subject 1—P2	30.852	0.183	64.03	12.59	0.0%
Subject 1—P3	31.321	0.258	68.23	10.58	0.0%
Subject 2—P1	26.943	0.301	61.96	14.89	20.0%
Subject 2—P2	30.311	0.428	91.87	31.32	23.3%
Subject 2—P3	30.472	0.319	57.77	7.57	13.3%
Subject 3—P1	28.455	0.598	63.42	13.59	13.3%
Subject 3—P2	30.760	0.672	95.71	19.86	53.3%
Subject 3—P3	31.849	0.316	83.25	15.22	73.3%
Subject 4—P1	29.320	0.288	78.44	15.22	46.7%
Subject 4—P2	31.100	0.155	72.40	13.04	83.3%
Subject 4—P3	31.562	0.309	83.33	18.77	90.0%
Subject 5—P1	27.574	0.128	69.42	11.59	0.0%
Subject 5—P2	31.117	0.207	79.57	15.42	0.0%
Subject 5—P3	31.788	1.359	75.42	13.59	0.0%
Subject 7—P1	28.492	0.150	71.93	11.41	0.0%
Subject 7—P2	30.998	0.117	68.87	9.99	0.0%
Subject 7—P3	31.297	0.463	67.57	8.06	0.0%
Subject 8—P1	29.646	0.278	69.17	5.00	3.3%
Subject 8—P2	27.947	0.551	74.60	18.79	0.0%
Subject 8—P3	28.322	1.366	71.90	16.63	0.0%
Subject 9—P1	29.288	0.482	62.23	8.24	0.0%
Subject 9—P2	30.783	0.211	77.93	13.52	0.0%
Subject 9—P3	30.288	0.249	73.07	10.48	0.0%
Subject 10—P1	28.160	0.448	65.23	8.88	0.0%
Subject 10—P2	29.317	0.252	63.60	13.06	0.0%
Subject 10—P3	30.363	0.309	70.13	9.99	0.0%

Data for subject 6 is missing due to sensor failure.

**Table 5:** Group Summary Results (All Subjects).

Metric	Phase 1 Mean ± SD	Phase 2 Mean ± SD	Phase 3 Mean ± SD
Skin Temp (°C)	28.143 ± 1.115	30.430 ± 0.999	30.899 ± 1.096
Skin Temp SD	0.306 ± 0.147	0.298 ± 0.177	0.604 ± 0.467
Heart Rate (bpm)	67.10 ± 5.67	76.82 ± 10.68	72.63 ± 7.29
Heart Rate SD	10.70 ± 3.17	16.30 ± 5.74	12.43 ± 3.99
HR Missing Rate (%)	8.33 ± 14.82	16.00 ± 26.97	17.66 ± 30.26

**Table 6:** Paired *t*-test Results (Comparison of HR between Condition P1 and P2).

Statistical Measure	Condition P1 (HR)	Condition P2 (HR)	Difference (D = P2 − P1)
Mean	65.50	78.20	12.70
Standard Deviation (SD)	6.78	13.06	15.65
Sample Size (n)	9	9	9

Test results, t-value = 2.566 *p*-value (Two-tailed, df = 8) = 0.029. Effect Size (Cohen’s d) = 0.812. The result indicates a statistically significant difference (*p* < 0.05) between Condition P1 and Condition P2, with a large effect size (Cohen’s d > 0.8).

## Data Availability

The datasets generated and analyzed during the current study are available from the corresponding author on reasonable request. The data are not publicly available due to privacy and ethical restrictions.
